# Recurrent femoral shaft fractures in a child with gnathodiaphyseal dysplasia: a case report

**DOI:** 10.1186/s12891-019-2464-9

**Published:** 2019-02-23

**Authors:** Takuma Kuroda, Ichiro Okano, Takatoshi Sawada, Satoshi Okamoto, Yuki Midorikawa, Tetsuya Tachibana, Toshio Yagi, Katsunori Inagaki

**Affiliations:** 1 Department of Orthopedic Surgery, Showa University Northern Yokohama Hospital, Chigasaki-chuo 35-1, Tsuzuki-ku, Yokohama-shi, Kanagawa 224-8503 Japan; 2 Department of Orthopedic Surgery, Ohta-Nisihinouchi hospital, 2-5-20 Nishinouchi, Koriyama, Fukushima, 963-8558 Japan; 3 Department of Orthopedic Surgery, Showa University School of Medicine, 1-5-8 Hatanodai Shinagawa-ku, Tokyo, 142-8555 Japan

**Keywords:** Gnathodiaphyseal dysplasia, Femoral shaft fractures, Recurrent fracture, External fixation, Intramedullary devise

## Abstract

**Background:**

Gnathodiaphyseal dysplasia (GDD) is an extremely rare autosomal dominant disease characterized by cemento-osseous lesions in the jawbones, bone fragility, and diaphyseal sclerosis of the tubular bones. Patients with GDD are prone to sustain fractures by minor accidents. Although over 80 cases have been reported, detailed information about the orthopedic treatment of the fractures is limited.

**Case presentation:**

A 9-year-old Japanese girl with a known history of GDD presented with pain and deformity in the left thigh after a minor fall. She had a displaced transverse fracture in the mid-shaft of the left femur and underwent a closed reduction and external fixation. In the 25th week after the initial surgery, she had another fracture in the left femur at one of the half-pin insertion sites. She underwent an external fixation again. After this operation, the patient sustained another refracture at the same fracture site and one supracondylar fracture at the distant site of the femur. The supracondylar fracture occurred without any triggering activity before beginning a weight-bearing exercise. The supracondylar fracture was successfully treated conservatively, but she sustained two more diaphyseal fractures at half-pin insertion sites one after another. She eventually underwent a revision surgery with a flexible intramedullary nail. At 3 months postoperatively, the fracture was healed and the patient maintained her ambulatory status without further refracture.

**Conclusions:**

Patients with GDD might have narrower safety ranges of biomechanical and physiological drawbacks, which are considered to be acceptable in ordinary cases. The choice of treatment should be aimed at minimizing these negative effects. We recommend intramedullary devise as the first-choice implant for the treatment of isolated femoral shaft fracture in GDD patients in this age group.

## Background

Gnathodiaphyseal dysplasia (GDD) is one of the extremely rare autosomal dominant diseases characterized by cemento-osseous lesions of the jawbones, bone fragility, and diaphyseal sclerosis of tubular bones [1]. The patients with GDD are prone to sustain fractures by minor accidents. To date, reports of approximately 80 affected individuals are available, and most of them have suffered fractures [1, 2]. Although the underlying mechanism of bone fragility has not been completely elucidated, recent investigations demonstrated that mutations in the ANO5 gene were associated with the pathogenesis of GDD [1]. There are several reports that discuss the treatment of facial bone lesions [3, 4]. However, detailed information about the orthopedic treatment of the fractures is limited. In this report, we present a case of recurrent femoral shaft fractures in a 9-year-old female patient with GDD after surgical treatment and address the challenges in fracture management in this rare and difficult condition.

## Case presentation

A 9-year-old Japanese girl with a known history of non-hereditary GDD presented with pain and deformity in the left thigh after a minor fall. The GDD was diagnosed by the facial bone developmental dysplasia and genetic examination when she was 3 years old. She had a history of multiple fractures of various sites, such as bilateral tibia, fibula, thoracic vertebrae, cervical vertebrae, and coccyx. All previous fractures had been successfully treated conservatively without complications. Her bone mineral density (BMD) measured using dual-energy X-ray absorptiometry at the previous hospital 2 years ago was lower than the age-adjusted average; her spine BMD was 0.493 g/cm^2^ and T-score was − 5.9.

**Fig. 1:**
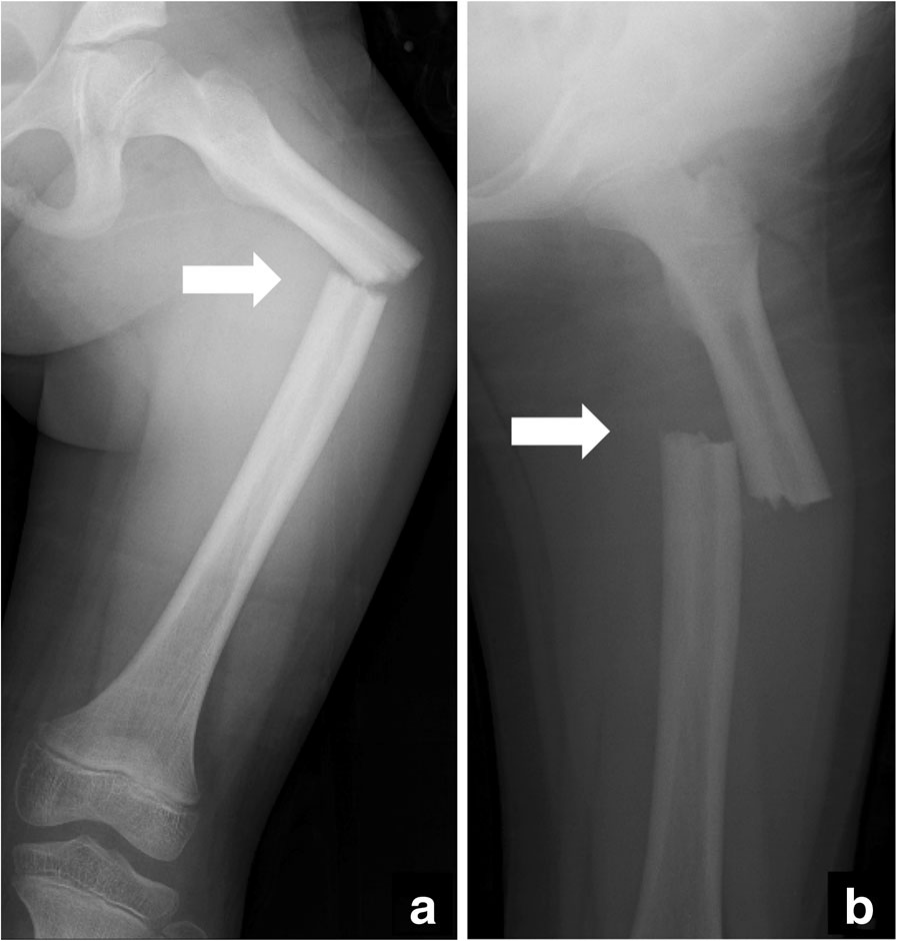
Radiographs of the left femur at the initial examination presenting a femoral shaft fracture (arrow). **a** the anteroposterior view, **b** the lateral view

**Fig. 2:**
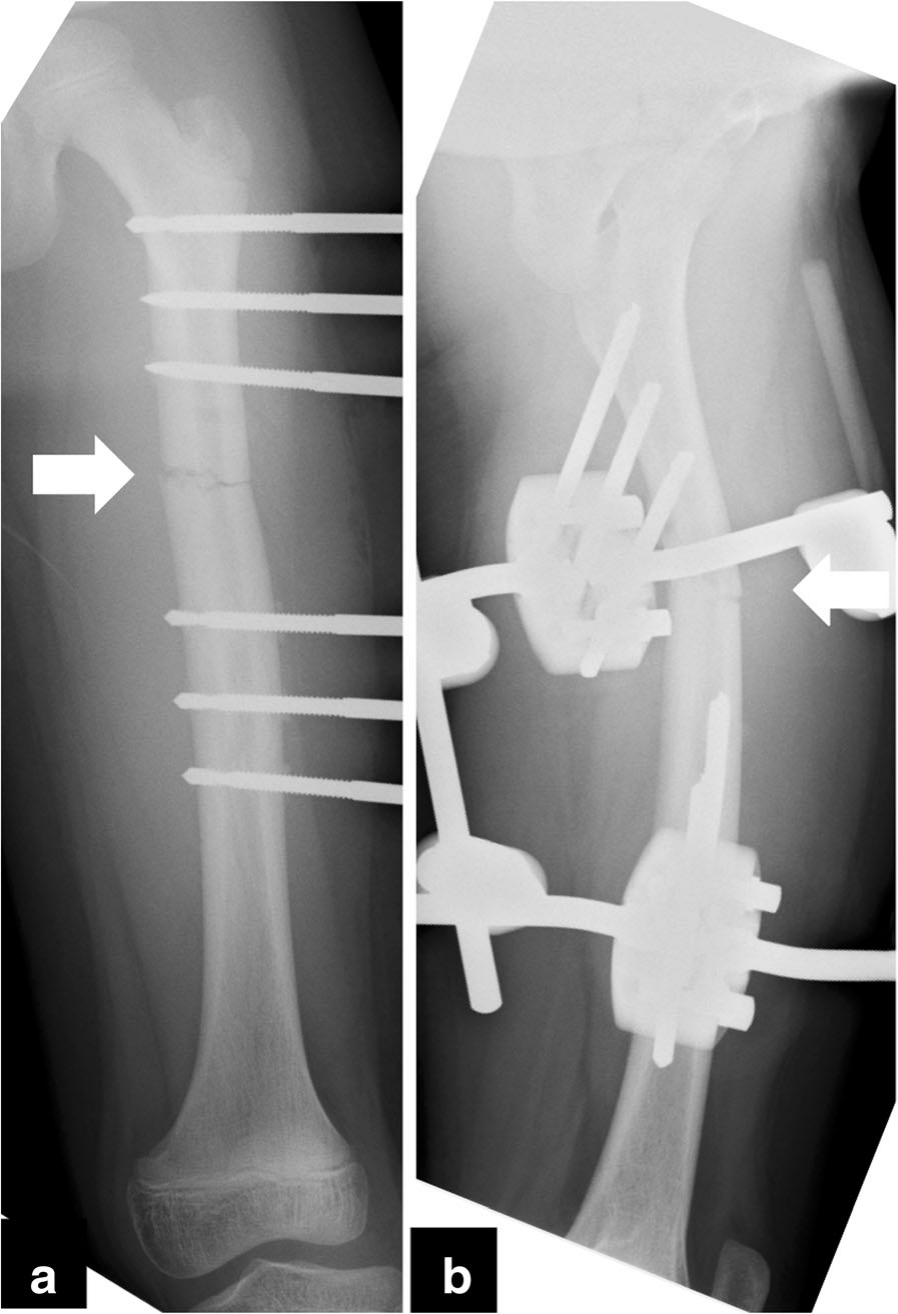
Radiographs of the left femur after the first closed reduction and external fixation with unilateral fixator system. **a** the anteroposterior view, **b** the lateral view. The fracture is reduced. (arrow)

**Fig. 3:**
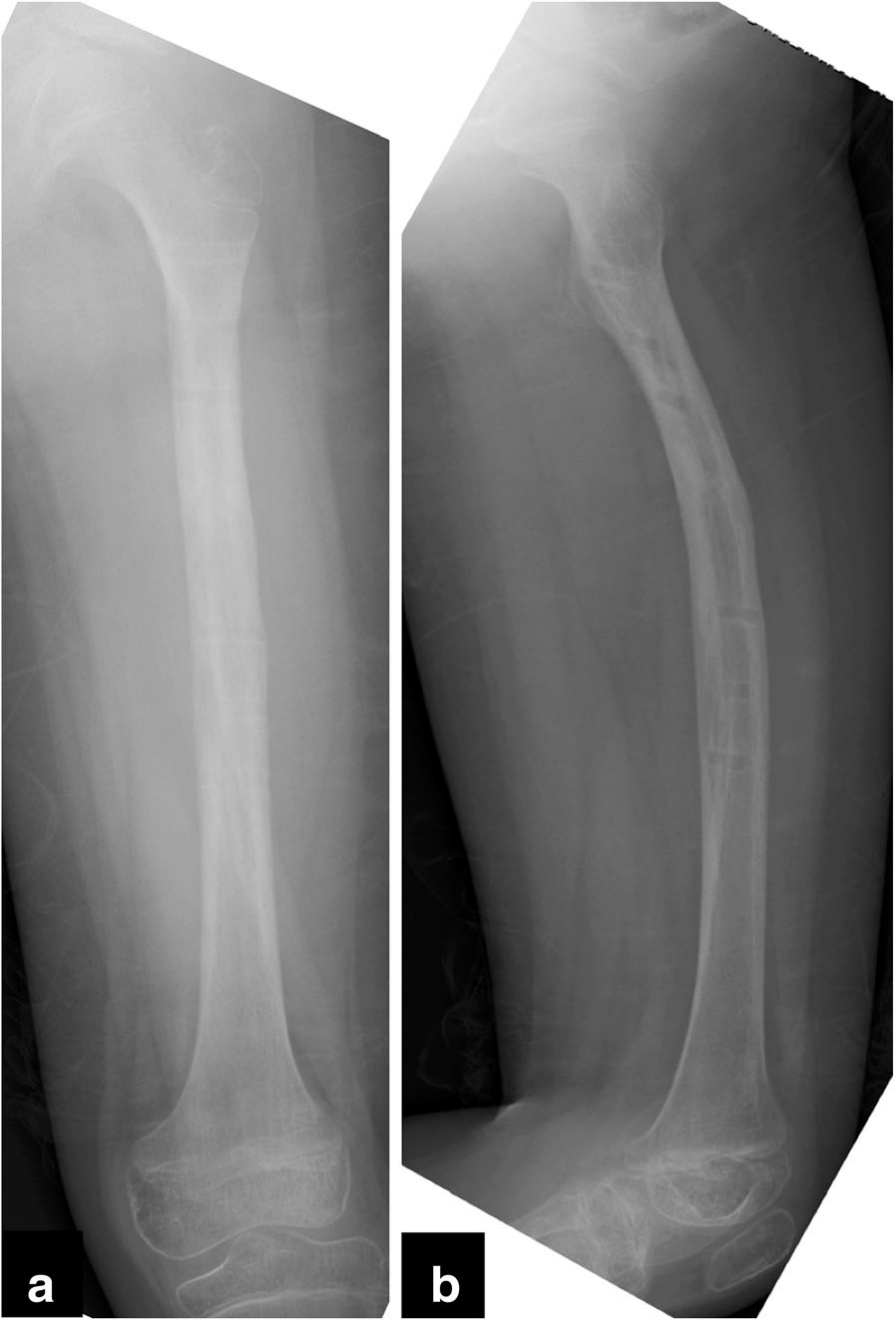
Radiographs of the left femur at the 12-week follow-up after the first operation. **a** the anteroposterior view, **b** the lateral view

At the initial radiologic examination, a displaced transverse fracture (32-D/4.1 in AO Pediatric Comprehensive Classification of Long Bone Fractures (AO-PCCF) [5]) in the mid-shaft of left femur was observed (Fig. 1). Repositioning of the fracture fragments was not successful using skin traction with external hanging weights. Thus, the patient underwent a closed reduction and external fixation by using a unilateral fixator system (Hoffmann II®, Stryker Corporation, Kalamazoo, MI) with 5-mm non-hydroxylapatite-coated half pins under general anesthesia 6 days after the injury (Fig. 2). The radiograph obtained at the 12-week follow-up showed a solid bony union at the fracture site, and the fixators were removed (Fig. 3). The patient was allowed to initiate and gradually advance weight bearing.

**Fig. 4:**
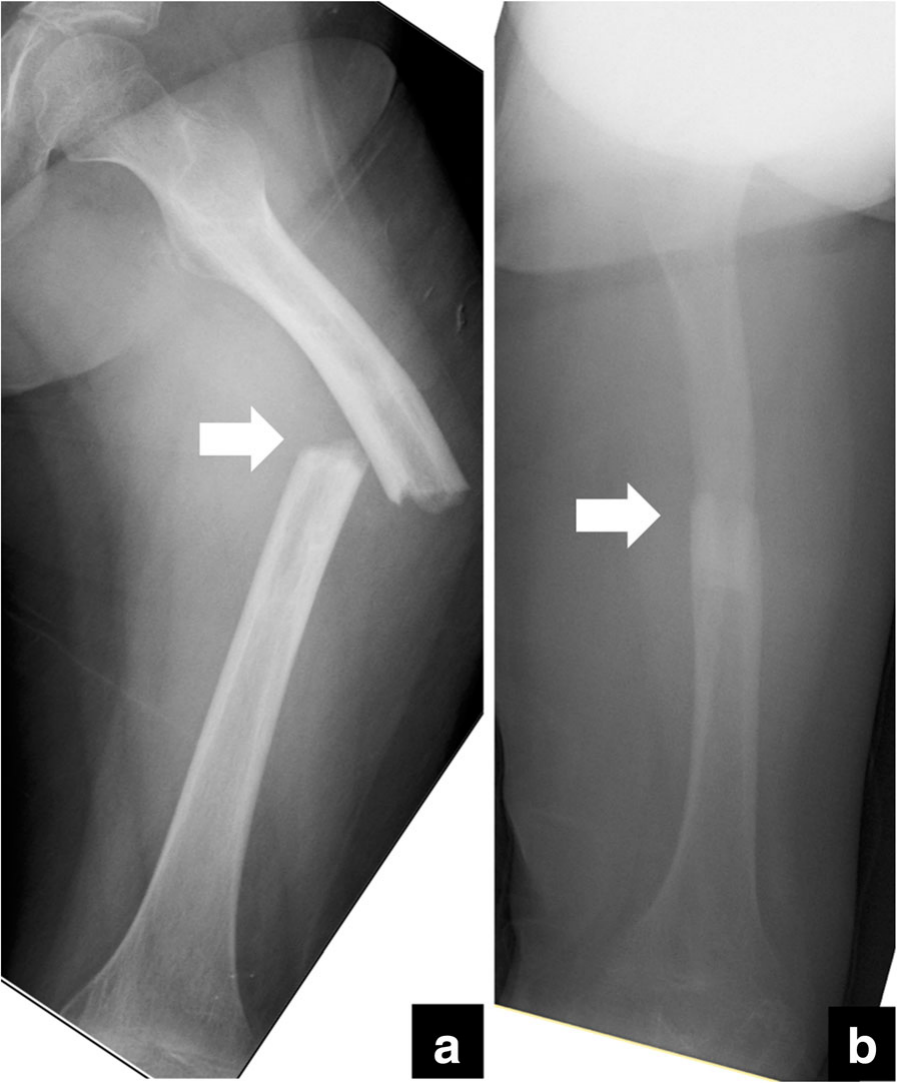
Radiographs of the left femur at the first refracture at a pin-site. (arrow) (**a**) the anteroposterior view, (**b**) the lateral view

**Fig. 5:**
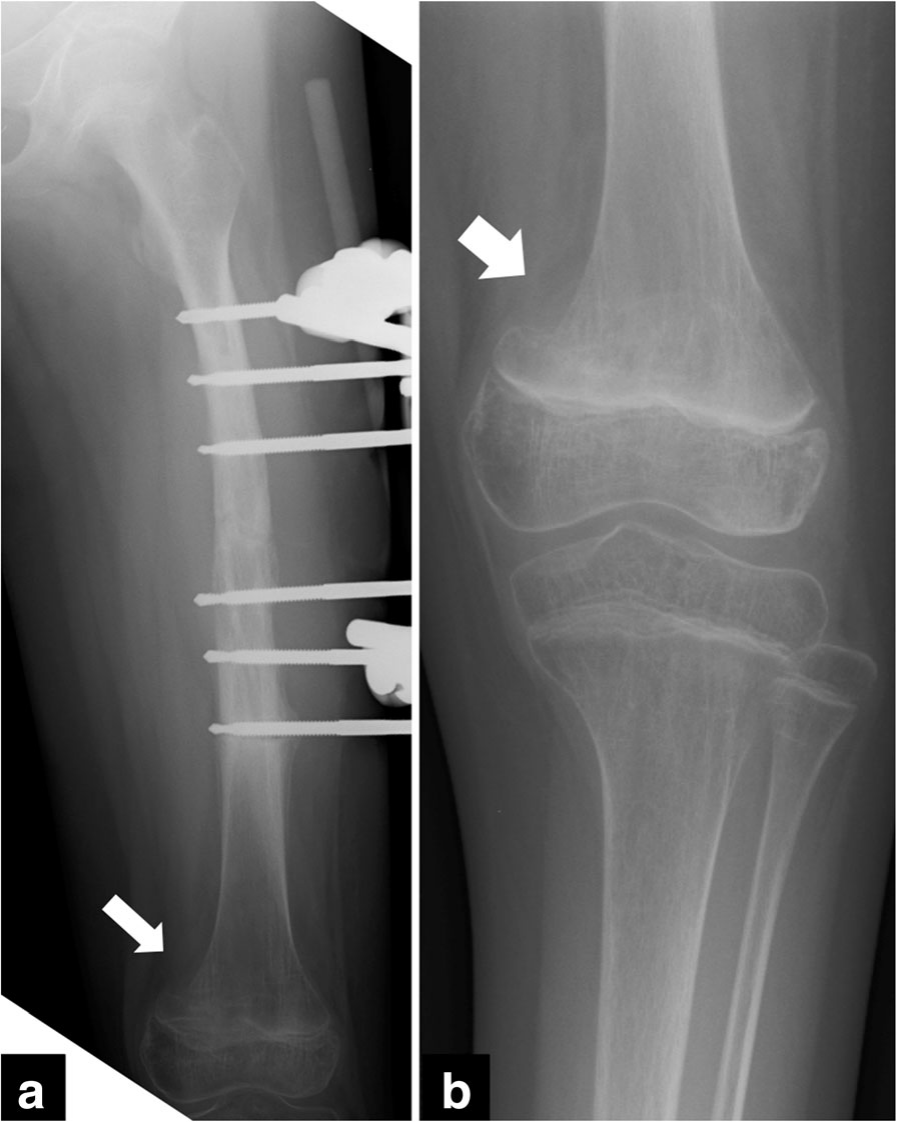
**a** A radiograph of the left femur presenting a supracondylar fracture at the distant site of the right femur. (arrow) (**b**) An enlarged image of the supracondylar fracture site

**Fig. 6:**
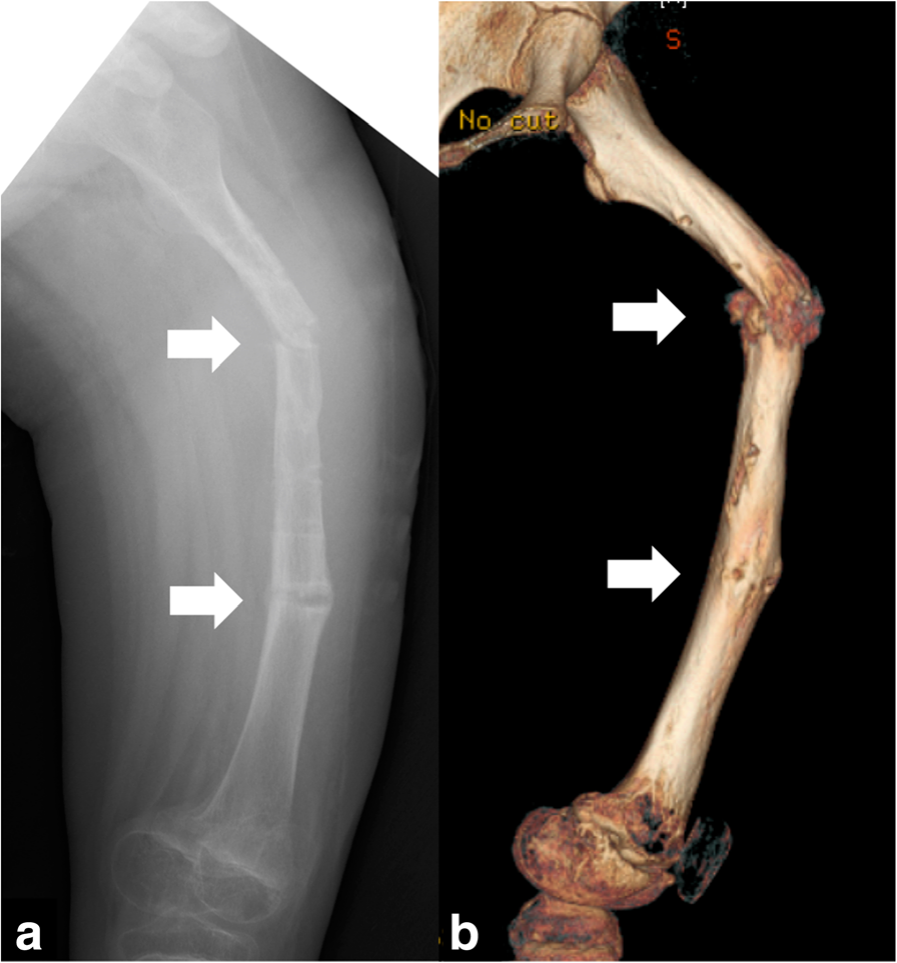
**a** A radiograph of the left femur presenting the two fractures at half-pin insertion sites. (arrows) (**b**) The three- dimensional reconstruction of the left femur presenting the two fractures at half-pin insertion sites. (arrows)

**Fig. 7:**
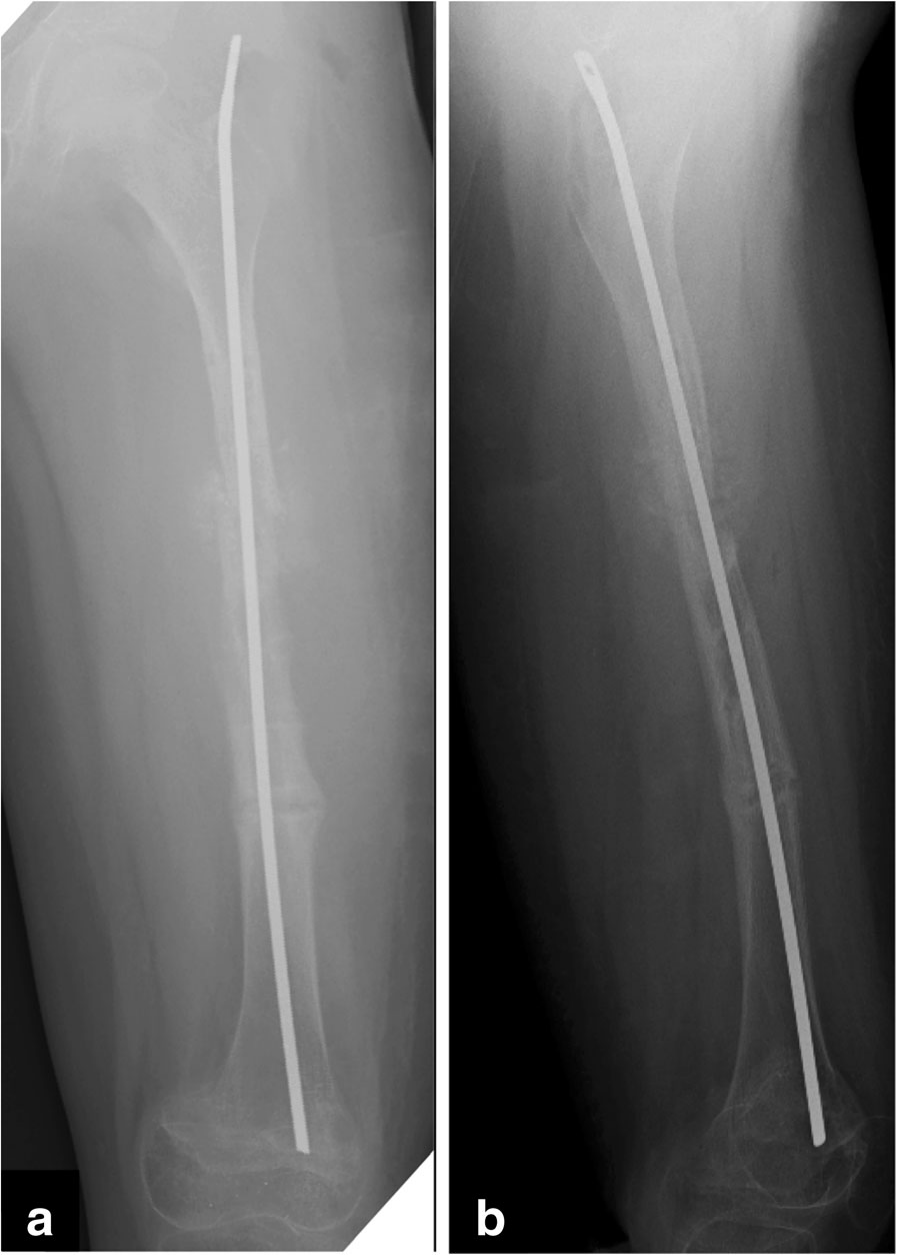
Radiographs of the left femur after the revision surgery with the Ender nail. **a** the anteroposterior view, **b** the lateral view

**Fig. 8:**
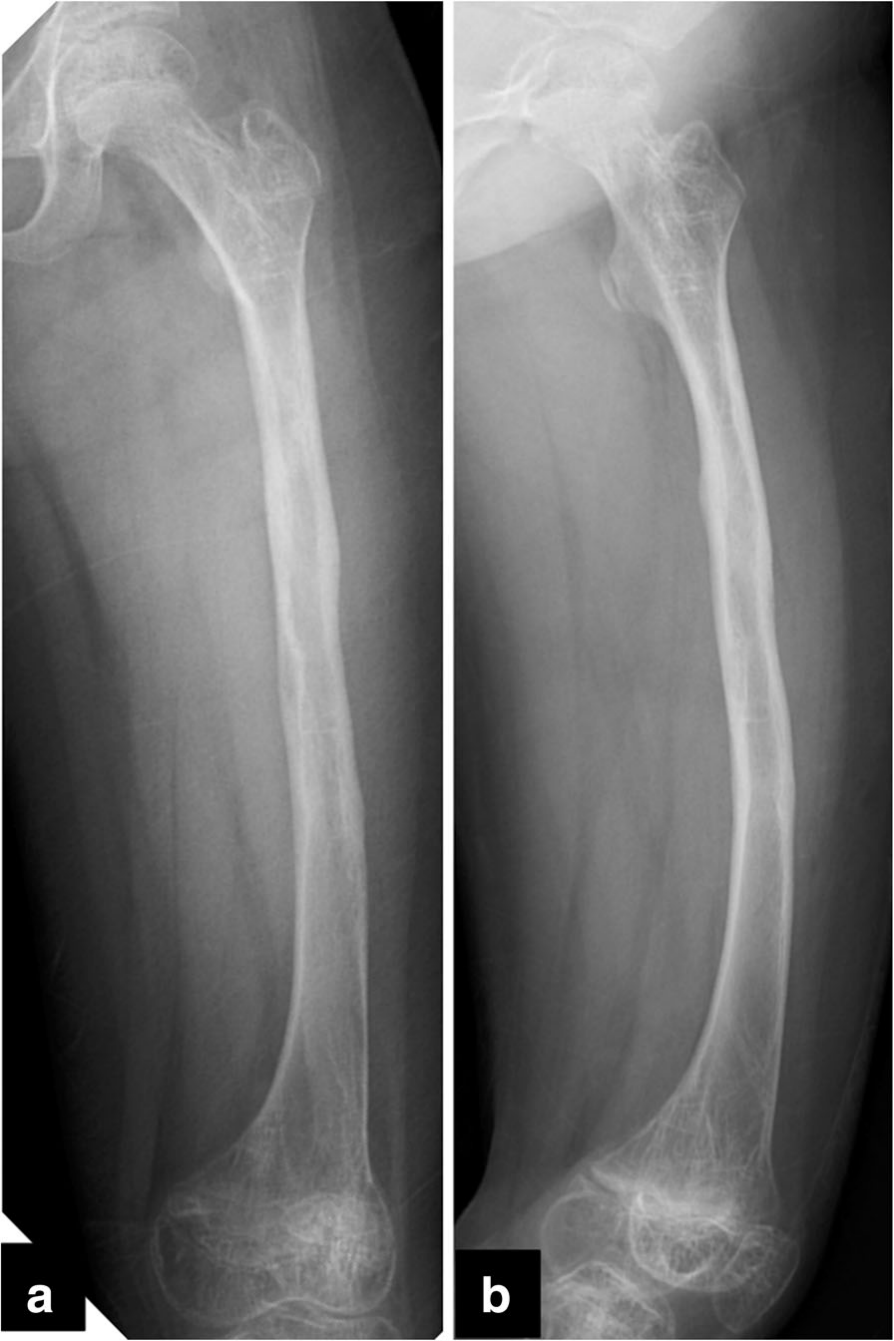
Radiographs of the left femur after the revision surgery after the removal of Ender nail (5-month after the nail insertion) showing solid bony fusion of all fractures. **a** the anteroposterior view, **b** the lateral view

At 25 weeks after the initial surgery, she suddenly felt severe pain in her left thigh while she was walking and was unable to walk further. Radiological examinations revealed another fracture in the left femur (32-D/4.1 in AO-PCCF) at one of the half-pin insertion sites (Fig. 4). She underwent an external fixation again. After this operation, the patient sustained a refracture (32-D/4.1 in AO-PCCF) at the same fracture site, followed by a supracondylar fracture (33-M/3.1 in AO-PCCF) at a distant site of the femur (Fig. 5) and two consecutive fractures at the half-pin insertion sites (Fig. 6). The supracondylar fracture occurred without any triggering activity before beginning weight-bearing exercise. The supracondylar fracture was successfully treated conservatively, but she sustained two more consecutive diaphyseal fractures (32-D/4.1 and 32-D/4.1 in AO-PCCF) at the half-pin insertion sites (Fig. 6). She eventually underwent a revision surgery for the diaphyseal fractures with an Ender nail (Ender nail®, MIZUHO Co., Ltd., Tokyo, Japan). Open reduction was not easily achieved owing to the fracture deformity and growing callus. Only one nail could be passed through it because the medullary canal was significantly narrowed due to diaphyseal sclerosis associated with GDD (Fig. 7). After this operation, the patient started early functional rehabilitation and was allowed to progress gradually to partial weight bearing following pain relief. The radiographs at 5-month follow-up showed a solid bony union at all the fracture sites, and the Ender nail was removed (Fig. 8). Three months after the implant removal, the patient maintained her ambulatory status without further refractures.

## Discussion and conclusion

We reported a case of a 9-year-old GDD patient with multiple recurrent fractures. To the best of our knowledge, no reports have been found regarding the detailed management of fracture in patients with GDD.

This patient was initially treated using a unilateral external fixator. However, the patient sustained multiple refractures in the fracture site as well as the half-pin insertion sites. Currently, more proportion of pediatric femoral shaft fractures are being treated surgically [6]. Several surgical options, including flexible intramedullary (IM) nail, solid IM nail, plate, and external fixation are available. At the time of the fracture, we adopted external fixation as the first-choice of treatment for the femoral shaft fracture in this age group. Previous studies showed that external fixation demonstrated results comparable with other fixation methods [7–9]. One of the major complications of external fixation is refracture after removal of the external fixator [8, 10–12]. In previous reports, rates for refracture varied from 0 to 21.6% [8, 12, 13]. In their case series, Carmichael et al. [8] described that longer time in fixator was one of the risk factors for refracture. Although they did not determine the clear threshold, on comparison with their average fixator duration (11.7 weeks), prolonged fixator use was not relevant in our patient as a cause for the first refracture (12 weeks).

Previous reports also mentioned two major sites for refractures associated with external fixation: original fracture sites and pin insertion sites. Refractures at pin insertion sites were less frequent [8]. We utilized 5-mm-diameter half pins, as recommended, for pediatric femur fractures [14]; however, our patient had two refractures at the half-pin insertion sites. A biomechanical study showed that the presence of a 10% bicortical defect of bone circumference is sufficient to produce a reduction in peak torque [15]. Cortical defects created by a 5-mm-diameter half pin are likely to exceed a 10% bicortical defect in this age group [16]; however, these defects do not cause fractures in most cases. The GDD patients might not tolerate relatively small cortical bone defects associated with external fixator pin insertion.

In our patient, a refracture occurred 13 weeks after the removal of the external fixation. A previous study showed that refractures usually occurred in the earlier phase, an average of 8 days (1–21 days) after fixation removal [13]. In their animal study, Albert et al. demonstrated early screw hole filling of the woven bone and regained bone strength at 4 weeks after screw removal [17]. Although one might argue that directly applying the results of non-human studies to patients can be an issue, 13 weeks appeared enough to restore bone strength to the safety range. The altered healing process in GDD patients might sustain pin holes at high risk for refractures in a longer time period. Considering these disadvantages, external fixation was not considered suitable for the closed femoral shaft fracture in patients with GDD.

After five refractures, our patient was successfully treated using the Ender nail®, a flexible IM nail (FIN). FlN is widely accepted as a reliable and safe option for treatment of pediatric femoral shaft fractures [18–20]. A recent meta-analysis showed that FIN had a superior effectiveness for pediatric femoral fractures compared to external fixation in terms of time to union, limb-length discrepancy, refracture rate, infection rate, pain, bursitis rate, and patient satisfaction [18]. Some reports showed that FIN allowed the patients to perform earlier functional exercises after operation [21].

The IM device is also used as a first-line treatment of long-bone fractures among patients with osteogenesis imperfecta (OI), which is a systemic osseous fragility disease similar to GDD [22]. For patients with OI, more rigid IM devices are preferred to obtain stability. Two types of IM devices are used for fractures in OI: elongating and non-elongating rods. Elongating rods have self-extending designs to follow bone growth. Recently developed elongating rods, such as Sheffield and Fassier–Duval medullary rods, have become the standard treatment for patients with OI [22]. El-Adl et al. reported that elongating rods are better than non-elongating rods, such as Kirschner wires or rush pins, with regards to mobility status, longevity, and incidence of complications requiring reoperations [23]. In a previous report on a case of GDD, although the authors did not provide the description in detail, they mentioned that the patient was treated using elongating rods (the Fassier–Duval medullary rods) [24]. Given that recurrent refractures in our patient were a result of treatment with EF, we recommend IM devices as the first choice for the treatment of femoral shaft fractures in GDD patients. In this case, we utilized the Ender nail as an IM device because elongating rods are not commercially available in our country and because other non-elongating IM devices such as rush pins and Kirchner wires only have limited size variations, which were not able to cover adequate lengths of the femur in our patient. It remains unclear whether fixation with a single Ender nail provides enough stability for patients with GDD. Considering the favorable results in OI, rigid and elongating rods may be a better option than non-elongating IM devices.

The non-ambulatory state is a known risk factor for secondary osteoporosis [25]. An increased risk of clinical fractures was observed among the non-ambulatory pediatric patients with chronic neuromuscular diseases [25, 26]. In our patient, a long period of postoperative immobilization and non-weight-bearing might have caused secondary osteoporosis, which led to a supracondylar femoral fracture after the 2nd refracture. Fragility fractures associated with immobilization and non-weight bearing after an isolated fracture in the normally ambulatory children have been rarely reported. Patients with GDD might be more sensitive to mechanical environmental change caused by non-weight bearing, which is not usually related to worse clinical outcomes. Given the higher incidence of refractures among GDD patients and the potential vulnerability to non-weight bearing, we believe the implants that allow early functional rehabilitation should be selected for the treatment of femoral fractures in GDD to prevent this vicious cycle of refractures.

To prevent recurrent fractures, pharmacological therapy for bone fragility may be considered. Among OI patients, bisphosphonate therapy is currently the most common medical treatment and has been reported to decrease the incidence of long-bone fractures [27]. On the other hand, little is known about medications for patients with GDD. Ghada et al. reported a case of GDD treated with a bisphosphonate [1]. They demonstrated that bisphosphonate therapy did improve BMD of the spine. However; no apparent benefit was observed in terms of the frequency or severity of fractures. We proposed bisphosphonate therapy for our patient but could not obtain the care-givers’ consent. They were mainly concerned about the potential adverse effects, particularly bisphosphonate-related osteonecrosis of the jaw, as the patient had GDD-associated lesions in the jawbone, which had required multiple surgical interventions. Therefore, further studies are needed to clarify the role of pharmacological therapy for GDD patients.

In summary, we suggest that patients with GDD might have narrower safety ranges of biomechanical (cortical defects by pin holes) and physiological (weight-bearing status) drawbacks, which are considered acceptable in ordinary cases. The choice of treatment should be aimed to minimize these negative effects, thus, within the available options, we recommend IM devise as the first-choice implant for isolated femoral shaft fracture in GDD patients in this age group.

## Abbreviations

AO-PCCF: AO Pediatric Comprehensive Classification of Long Bone Fractures OI osteogenesis imperfecta
BMD: bone mineral density
FIN: flexible intramedullary nail
GDD: Gnathodiaphyseal dysplasia
IM: intramedullary
